# Nanopore and EPIC array profiling unmask an atypical non-midline H3 K27-altered glioma: a case report

**DOI:** 10.3389/fonc.2026.1897287

**Published:** 2026-08-12

**Authors:** Michela Buonaiuto, Pasqualina Giordano, Alfredo D’Avino, Ornella Affinito, Mariella Cuomo, Marta Sabbarese, Angela Viggiano, Francesca Maria Giugliano, Alberto Negro, Federico Frio, Giuseppe Catapano, Lorenzo Chiariotti, Rosa Della Monica

**Affiliations:** 1 CEINGE-Advanced Biotechnologies “Franco Salvatore”, Napoli, Italy; 2 U.O.C. Oncologia, Ospedale del Mare, Azienda Ospedaliera Locale (ASL NA1) Centro, Napoli, Italy; 3 U.O.C. Anatomia Patologica, Ospedale del Mare, Azienda Ospedaliera Locale (ASL NA 1) Centro, Napoli, Italy; 4 AREA Science Park, Trieste, Italy; 5 Department of Molecular Medicine and Medical Biotechnologies, University of Napoli “Federico II”, Naples, Italy; 6 U.O.C. Radioterapia, Ospedale del Mare, ASL NA1 Centro, Napoli, Italy; 7 U.O.C. Neuroradiologia, Ospedale del Mare, Azienda Ospedaliera Locale (ASL NA1) Centro, Napoli, Italy; 8 U.O.C. Neurochirurgia, Ospedale del Mare, Azienda Ospedaliera Locale (ASL NA1) Centro, Napoli, Italy

**Keywords:** epigenomic profile, K27M adult glioma, methylation brain tumor classifier, nanopore sequencing, non-midline incisional hernia

## Abstract

We report a rare case of Diffuse Midline Glioma, H3K27-altered, arising in a non-midline fronto-temporo-insular location in a 48-year-old adult, initially mimicking Glioblastoma, IDH-wildtype, based on radiology and histopathology. Integrated molecular diagnostics, including genome-wide DNA methylation profiling and Sanger sequencing, confirmed an H3.3 K27M mutation, while Nanopore-based methylation profiling provided a rapid and fully concordant classification, demonstrating the feasibility of real-time epigenomic diagnostics in clinical practice. H3K27M-mutant diffuse non-midline gliomas (DNMG) are extremely rare, with only ~39 cases reported in the literature, most commonly involving the temporal lobe and associated with poor prognosis. Our case highlights that DNMGs can present in adults and in previously unreported hemispheric locations, underscoring the importance of integrated molecular testing not only for accurate diagnosis but also to guide patient management and enable enrollment in potential targeted therapies. Molecular profiling thus represents a critical tool for personalized treatment planning and prognostic assessment in these uncommon gliomas.

## Introduction

Pediatric-type diffuse gliomas include low- and high-grade families. According to WHO 2021 (1), the class of pediatric-type diffuse high-grade glioma include: Diffuse midline glioma, H3 K27-altered; Diffuse hemispheric glioma, H3 G34-mutant; Diffuse pediatric-type high-grade glioma, H3/IDH wildtype; and Infant-type hemispheric glioma. This classification replaced WHO 2016, recognizing additional molecular alterations beyond H3 K27M in diffuse midline gliomas (2).

Diffuse midline gliomas H3 K27-altered are driven most commonly by H3K27M mutations but may also involve EZHIP overexpression or other alterations. H3K27M disrupts PRC2 activity, causing loss of H3K27me3, de-repression of normally silenced genes, epigenetic remodeling, and increased stemness (3, 4). EGFR mutations or amplifications can co-occur (5). These tumors are usually infiltrative, high-grade, Central Nervous System (CNS) WHO grade 4, pediatric, and located predominantly in brainstem, thalamus, or spinal cord, with poor prognosis (6–8).

Although diffuse midline gliomas are, by definition, expected to arise in midline structures, a small number of H3K27M-mutant diffuse gliomas occurring in non-midline cerebral locations have been described, mainly in adults (9–11). To date, approximately 39 cases have been reported in the literature. These tumors most frequently involve the temporal lobe, but have also been described in the frontal, parietal, and occipital lobes, and generally exhibit an aggressive clinical course. The main clinicopathological, molecular, and outcome characteristics of the published cases are summarized in **Table 1** (10–13, 16–24).

**Table 1:** Characteristics of atypical DMGs with H3K27M mutation from the previous publications.

Case	Reference	Tumor location	Histological grade	*ATRX/* *TP53*	Other molecular alterations:	Methylome classification	Follow-up months
1	Guidara S, et al. (12)	Temporo-polar region	3	*ATRX*: mutated with Protein loss TP53: WT	*PDGFRA* mutated: (S716G)	Diffuse midline glioma H3K27M –mutant	18
2	Guidara S, et al. (12)	Temporal lobe	3	ATRX: Protein loss TP53: Mutated (R175H)	*PDGFRA/KIT* amplification KRAS mutated (Q61R)	Diffuse midline glioma H3K27M –mutant	3
3	Sturm D, et al. (13)	Temporal lobe	4	ATRX: no data TP53: wt	No data	No data	5
4	Fontebasso AM, et al. (14)	Temporo- parietal lobe	High	ATRX: no data TP53: Mutated	*PDGFRA* mutated	No data	No data
5	Mackay A et al. (15)	Frontal lobe	4	*ATRX* mutated (V278fs) TP53: Mutated (I163fs)	*PDGFRA* Mutated (C235Y) *PDGFRA* amplification	No data	15
6	Mackay A et al. (15)	Temporal lobe	4	*ATRX* mutated (K356fs) TP53: Mutated (G245S)	*PDGFRA* mutated (D576G) *PTEN* Mutated (M270fs-)	No data	0.1
7	Mackay A, et al. (15); Sturm D, Witt H, Hovestadt V, et al. (13); Schwartzentruber J, et al. (16)	Temporo- parietal lobe	4	ATRX: Wildtype TP53: Mutated (R213*)	*PDGFRA* Mutated (K385M)	GBM-K27 No match	6
8	Mackay A, et al. (15); Sturm D, Witt H, Hovestadt V, et al. (13);	Temporal lobe	4	ATRX: No data TP53: Mutated	No mutations	GBM-K27 No match	8
9	Mackay A, et al. (15)	Cerebrum	3	ATRX: No data TP53: Mutated	No mutations	GBM-K27	2,9
10	Mackay A, et al. (15)	Cerebrum	3	*ATRX* mutated (D964fs) TP53: Mutated (R273C)	*FGFR1* mutated (N455K)	No data	8,0
11	Mackay A, et al. (15)	Cerebrum	4	No data	*PDGFRA* amplification	No data	4,8
12	Mackay A, et al. (15)	Cerebrum	4	ATRX: Wildtype TP53: Mutated (R273H)	*PDGFRA* amplification	No data	9,6
13	Mackay A, et al. (15)	Cerebrum	4	No data		No data	4,8
14	Mackay A, et al. (15)	Cerebrum	3	No data		No data	4,8
15	López G, et al. (17)	Insular cortex	2	ATRX: Protein retained *ATRX* mutated Q2194del TP53: WT	*EPB41L2-BRAF* fusion	No data	No data
16	Sturm D, et al. (13)	Parietal and occipital lobe	4	No data	No data	Diffuse midline glioma H3K27M –mutant	No data
17	Sturm D, et al. (13)	Frontal lobe	4	No data	No data	Diffuse midline glioma H3K27M -mutant	No data
18	Wang L, et al. (10)	Temporal lobe	3	ATRX: Protein retained TP53: Mutated	No data	No data	32,8
19	Wang L, et al. (10)	Frontal lobe	3	ATRX: Protein retained TP53: Mutated	No data	No data	6,6
20	Chia N, Wong A, Teo K, et al. (11)	Fronto- parietal lobe	4	ATRX: Protein loss TP53: Mutated (5-10%)	No data	No data	42
21	Valério E, et al. (18)	Temporal lobe	3	*ATRX*: Protein loss with no mutation TP53: Mutated (R273H)	PIK3CA mutated (E545K)	Diffuse midline glioma, H3K27M -mutant	11
22	Onishi S, Ohba S, Kuraoka K, et al. (19)	Temporal lobe	4	ATRX: Protein loss TP53: Mutated (P278T)	loss of PTEN	No data	13
23	Qiu T, Chanchotisatien A, Qin Z, et al. (20)	Temporal lobe	No data	No data	No data	No data	No data
24	Qiu T, Chanchotisatien A, Qin Z, et al. (20)	Occipital lobe	No data	No data	No data	No data	No data
25-28	Huang T, Garcia R, Qi J, et al. (21)	Cerebral hemisphere	No data	No data	No data	No data	No data
29	Odia Y, Hall MD, Cloughesy TF, et al. (22)	Frontal lobe	No data	No data	No data	No data	No data
30	Odia Y, Hall MD, Cloughesy TF, et al. (22)	Parietal lobe	No data	No data	No data	No data	No data
31	Odia Y, Hall MD, Cloughesy TF, et al. (22)	Temporal lobe	No data	ATRX mutated	PIK3CA mutated	No data	No data
32	Odia Y, Hall MD, Cloughesy TF, et al. (22)	Frontal lobe	No data	No data	No data	No data	No data
33	Odia Y, Hall MD, Cloughesy TF, et al. (22)	Frontal lobe	No data	ATRX mutated TP53 mutated	No data	No data	No data
34	Donev K, Sundararajan V, Johnson D, et al. (23)	Temporal lobe	High	ATRX: WT TP53: WT	No mutations in *EGFR, FGFR1 PDGFRA, BRAF, ACVR1* and *PIK3CA*	Diffuse midline glioma, H3K27M -mutant	84
35	Donev K, Sundararajan V, Johnson D, et al. (23)	Parietal lobe	High	ATRX: Protein loss ATRX mutated (D1940Vfs*14) TP53: mutated (E271V)	No mutations in *EGFR, FGFR1 PDGFRA, BRAF, ACVR1* and *PIK3CA*	Diffuse midline glioma, H3K27M -mutant	14
36	Venneti S, Santi M, Felicella MM, et al. (24)	Cerebrum	4	No data	No data	No data	No data
37	Venneti S, Santi M, Felicella MM, et al. (24)	Frontal lobe	4	No data	No data	No data	No data
38	Venneti S, Santi M, Felicella MM, et al. (24)	Occipital lobe	4	No data	No data	No data	No data
39	Venneti S, Santi M, Felicella MM, et al. (24)	Cerebrum	4	No data	No data	No data	No data

Despite these reports, the biological significance, optimal diagnostic workflow, and clinical management of diffuse non-midline H3K27-altered gliomas remain poorly defined because of their exceptional rarity. Furthermore, atypical anatomical locations may lead to an initial diagnosis of glioblastoma or other diffuse gliomas if comprehensive molecular profiling is not performed.

We report a rare case of an adult diffuse non-midline H3K27-altered glioma arising in the fronto-temporo-insular region, highlighting the importance of integrated histopathological and molecular evaluation including genome-wide DNA methylation profiling and rapid nanopore-based methylation analysis for accurate diagnosis, prognostic stratification, and personalized patient management.

## Results

### Case description

A 48-year-old Caucasian woman presented with sudden-onset, persistent headache. No secondary causes, including medication use or underlying systemic disease, were identified. Her past medical history was notable for laparoscopic cholecystectomy for cholelithiasis, gastroesophageal reflux disease, dyslipidemia, and lactose intolerance. She was premenopausal and had no focal neurological deficits on examination.

**Figure 1:**
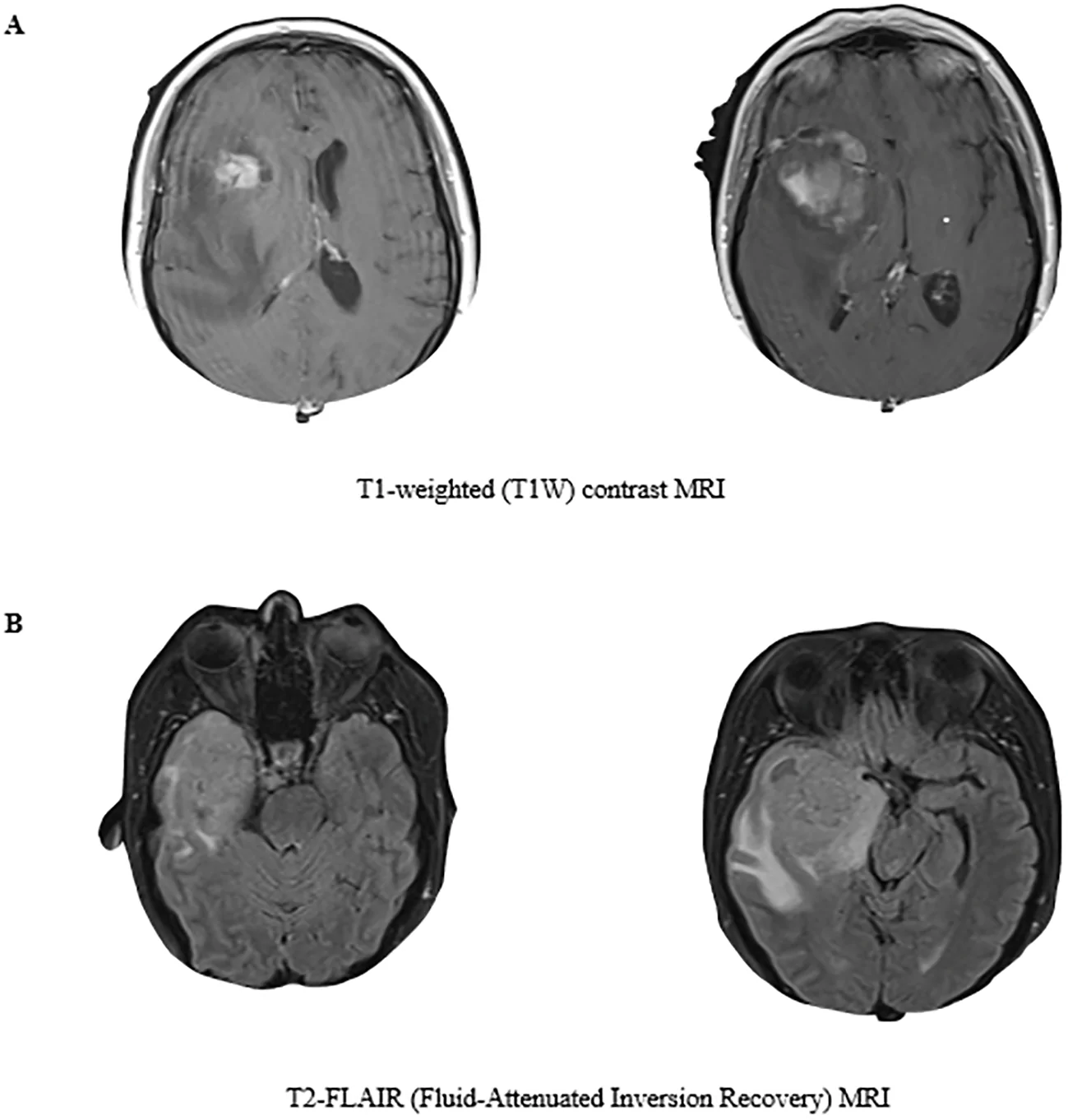
MRI pre surgery: **(A)** T1 contrast MRI images **(B)** T2-FLAIR MRI image.

After conservative management failed to alleviate her symptoms, a non-contrast brain computed tomography (CT) scan was performed, revealing a right intra-axial lesion involving the fronto-capsular region. Subsequent contrast-enhanced magnetic resonance imaging (MRI) demonstrated a large (51 × 42 × 52 mm) heterogeneous mass encompassing cortical, subcortical, and deep structures of the right fronto-temporo-insular region. The lesion appeared hypo intense on T1-weighted images and showed heterogeneous T2-weighted images, with fluid–fluid levels suggestive of cystic or necrotic components, and irregular contrast enhancement. Perfusion imaging revealed elevated relative cerebral blood volume, consistent with hypervascularity (**Figure 1**).

The lesion was located in a critical region, with encasement of the right middle cerebral artery and abutment of major vascular structures, including the carotid apex. Extensive surrounding vasogenic edema was observed, accompanied by secondary right uncal herniation and a contralateral midline shift of approximately 9 mm. Collectively, these imaging features were highly suggestive of a primary high-grade intra-axial glioma.

The patient subsequently underwent microsurgical resection. Intraoperatively, the lesion appeared firm, highly vascularized, and poorly demarcated from the surrounding brain tissue, with an indistinct cleavage plane, consistent with a high-grade glioma. Fluorescein guidance and an electrified suction device were used, and a subtotal resection was performed using bipolar coagulation, suction, and an ultrasonic aspirator, with the aim of achieving the maximal safe extent of resection while minimizing the risk of neurological morbidity.

The patient underwent subtotal surgical resection, followed by an uneventful postoperative course, with good clinical and neurological recovery and no relevant surgery-related complications. Adjuvant radiotherapy was subsequently administered. Treatment was delivered using volumetric modulated arc therapy (VMAT) with 6-MV photons and daily image-guided radiotherapy verification by cone-beam CT, using an advanced six-degree-of-freedom positioning system (HexaPOD). The surgical cavity and residual tumor in the right temporopolar and mesial temporal region received a total dose of 60 Gy in 30 fractions of 2 Gy each. At the latest follow-up in July 2026, the patient remained in good clinical condition. Based on the molecular profile of the tumor, she was enrolled in a clinical trial investigating ONC201 in patients with H3 K27-altered diffuse glioma. She is currently receiving treatment within the clinical trial and remains under regular clinical and radiological follow-up.

### Histological findings

**Figure 2:**
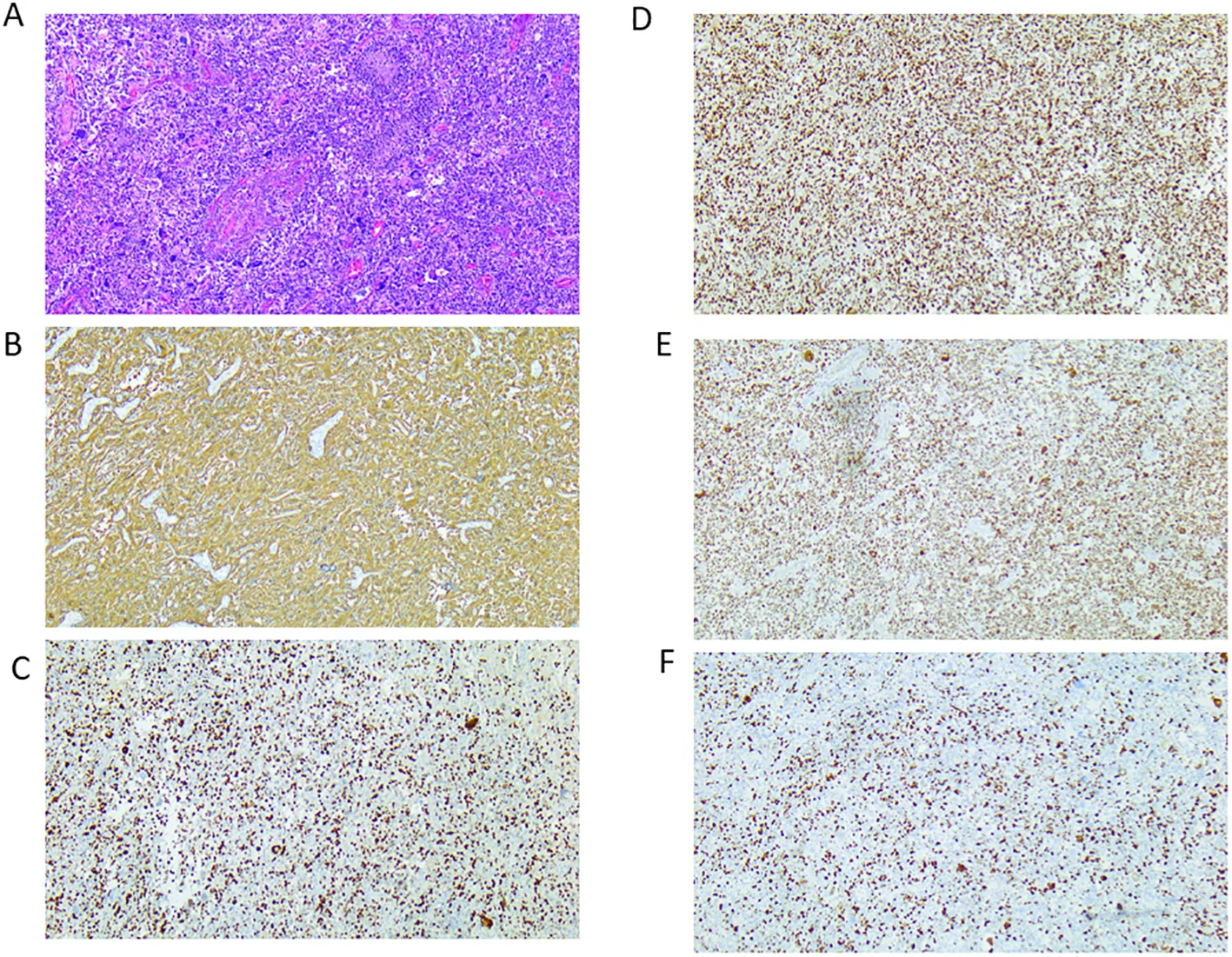
Histopathological diagnosis: **(A)** Hematoxylin and eosin staining demonstrates the histological hallmarks of a high-grade glial tumor, including markedly increased cellularity, pronounced nuclear atypia with occasional multinucleated cells, pseudo-palisading necrosis, prominent microvascular proliferation, and numerous mitotic figures, consistent with aggressive astrocytic neoplasia. **(B)** IHC for GFAP indicating a glial neoplasia. **(C)** IHC for OLIG-2. **(D)** IHC for ATRX. **(E)** IHC for Tp53. **(F)** IHC Ki67 >70%.

Histological examination revealed fragments of a highly cellular glial neoplasm with marked nuclear atypia and pronounced pleomorphism. The tumor was composed of large atypical cells with bizarre, enlarged, hyperchromatic nuclei, occasionally multinucleated, interspersed with areas of necrosis, including focal “pseudopalisading” patterns. Prominent microvascular proliferation was also observed. The mitotic activity was markedly elevated, with approximately 40 mitoses per 10 high-power fields, including atypical mitotic figures highlighted by phospho-histone H3 immunohistochemistry. Immunohistochemical analysis showed tumor cell positivity for GFAP, Olig-2, and p53, with retained ATRX expression. Staining for cytokeratin (CAM 5.2) was negative. The Ki-67 proliferation index was significantly increased, with approximately 70% of tumor nuclei showing positivity (**Figure 2**). Taken together, the morphologic and immunophenotypic features were consistent with a high-grade diffuse astrocytic neoplasm, consistent with glioblastoma, IDH-wildtype, giant cell subtype (CNS WHO grade 4), in concordance with the neuroradiological and intraoperative findings.

### Molecular findings

**Figure 3:**
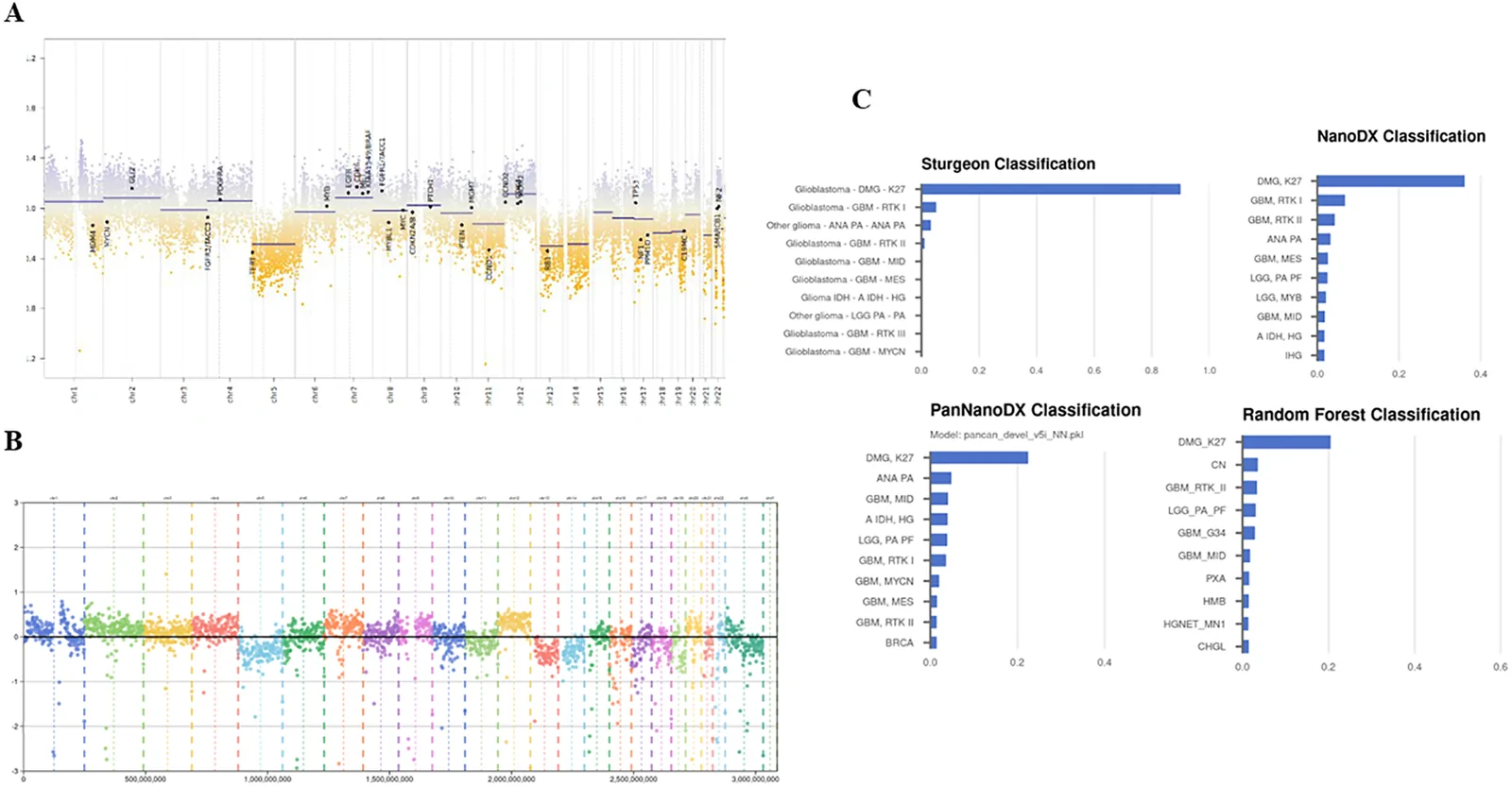
Molecular diagnosis: **(A)** CNV profile derived from Illumina EPIC methylation array data. **(B)** CNV profile derived from Oxford Nanopore sequencing data. **(C)** Methylation-based tumor classification performed using the ROBIN framework on Nanopore-derived data with multiple classifiers, all concordantly supporting a diagnosis of Diffuse midline glioma, H3 K27-altered. Sturgeon classified the sample with a calibrated score of 0.89; NanoDX (Capper et al. neural network) with a calibrated score of 0.36; PanNanoDX with a calibrated score of 0.22; and the Random Forest classifier with a calibrated score of 0.56.

Genome-wide DNA methylation profiling was performed using the Illumina EPIC 950K array. Copy number variation (CNV) analysis derived from array data demonstrated chromosomal deletions with loss of material involving chromosomes 5, 13, and 14 (**Figure 3A**). These alterations were independently confirmed by CNV analysis obtained from Nanopore sequencing data (**Figure 3B**).

Methylation-based classification using the Heidelberg CNS tumor classifier (DKFZ) yielded a high-confidence match (calibrated score: 0.98) with the methylation class corresponding to diffuse midline glioma, H3K27-altered (**Figure 3C**).

To further confirm tumor subtyping, mutational analysis of histone H3 variants was performed. Sanger sequencing identified the H3.3 K27M mutation, enabling definitive classification as diffuse midline glioma, H3K27M-mutant, CNS WHO grade 4.

Targeted next-generation sequencing (NGS) revealed a variant of uncertain significance in TP53 (c.823T>C p. Cys275Arg) and a pathogenic variant in KRAS (c.35G>C, p. Gly12 Ala).

To complement array-based methylation profiling and assess rapid diagnostic feasibility, whole-genome Nanopore sequencing. Genomic DNA extracted from formalin-fixed paraffin-embedded (FFPE) tumor tissue was used for amplification-free whole-genome sequencing using the Oxford Nanopore Technologies ligation sequencing protocol (SQK-LSK114). Sequencing was performed on a PromethION 2 Integrated platform (FLO-PRO114M flow cell). Real-time high-accuracy (HAC) basecalling was carried out using Dorado v7.11.2 (MinKNOW v25.09.16) and dna_r10.4.1_e8.2_400bps_hac@v5.2.0 as basecalling model with simultaneous modified base detection (5-methylcytosine and 5-hydroxymethylcytosine in CpG contexts). Reads were aligned to the human reference genome hg38 using the integrated MinKNOW alignment workflow. The minimum quality threshold for passed reads was set to Q9. The sequencing run was performed for 72 hours. After the sequencing run, approximately 9.1 million reads were generated. A total of 816,593 reads passed the quality filter, corresponding to 253.5 million high-quality bases, with an estimated read N50 of 490 bp. A total of 328,802 mapped reads were available for downstream methylation analysis. Global estimated genome coverage was 0.03× (target coverage 0.05×). The relatively low coverage, together with the short read N50, reflects the fragmented nature of FFPE-derived DNA. Nevertheless, sufficient informative CpG sites were obtained to allow reliable methylation-based classification. Methylation-based tumor classification was performed using ROBIN (25) v0.1.7 with the rCNS2 reference panel. The analysis incorporated four independent classifiers: Sturgeon, NanoDX, PanNanoDX and Random Forest. All classifiers consistently identified the sample as a diffuse non-midline glioma, H3 K27-altered, with the highest confidence provided by the Sturgeon classifier (score = 0.8995), followed by NanoDX (0.361), PanNanoDX (0.224) and Random Forest (18.13). Methylation-based classification was performed retrospectively using the complete set of BAM files generated during the sequencing run. Consequently, the exact sequencing elapsed time at which a stable classification was first achieved was not recorded. Once ROBIN was launched on a workstation equipped with an NVIDIA RTX A6000 GPU (48 GB VRAM) and 500 GB RAM, methylation-based classification was completed in within 5 minutes.

All raw data have been made publicly available. Illumina EPIC methylation array data have been deposited in the Gene Expression Omnibus (GEO) under accession GSE338747, and Oxford Nanopore sequencing data have been deposited in the NCBI Sequence Read Archive (SRA) under accession SRR39638246. Both datasets are linked through NCBI BioProject PRJNA1495515.

## Discussion

Diffuse midline glioma, H3K27-altered, is defined by alterations affecting histone H3 lysine 27 and is classified as CNS WHO grade 4 regardless of histologic features. These tumors predominantly arise in pediatric patients and typically involve midline structures such as the pons, thalamus, or spinal cord. In contrast, our case occurred in a 48-year-old adult and involved a fronto-temporo-insular (non-midline) location, representing an atypical clinical and anatomical presentation. The main clinical, radiological, histopathological, and molecular differences between the present case and the typical features of H3K27-altered diffuse midline glioma reported in the literature are summarized in **Table 2**. Although H3K27M-mutant gliomas are strongly associated with midline sites, increasing evidence indicates that this molecular alteration can also occur in atypical hemispheric locations and in adult patients. A recent institutional review identified only 2 patients (4%) with non-midline tumors among 51 H3K27M-mutant diffuse gliomas. Including previously reported cases, 39 non-midline H3K27M-mutant diffuse gliomas have been described to date. These tumors most frequently involve the temporal lobe, tend to affect adolescents and adults, and are associated with a poor prognosis, with a reported median overall survival of approximately 10.3 months. Molecularly, diffuse gliomas harboring H3K27 alterations frequently show concurrent TP53 (74%), ATRX (71%), and PDGFRA (44%) alterations, and DNA methylation profiling demonstrates clustering within or close to the reference group of midline H3K27M diffuse gliomas, supporting a shared molecular identity despite their atypical anatomical location.

**Table 2:** Comparison between the clinicopathological features of typical diffuse midline glioma, H3K27-altered, and the present atypical case.

Feature	Typical DMG, H3K27-altered	Present case
Age	Predominantly children and adolescents	48-year-old adult
Location	Midline (pons, thalamus, spinal cord)	Right fronto-temporo-insular (non-midline)
MRI findings	Diffuse infiltrative midline lesion, variable enhancement	Large heterogeneous hemispheric mass with irregular contrast enhancement, cystic-necrotic components
Histology	Diffuse infiltrating glioma, high-grade morphology	Microvascular proliferation, pseudopalisading necrosis, highly cellular diffuse astrocytic neoplasm with marked pleomorphism, histological features consistent with diffuse glioma, WHO grade 4
H3K27 alteration	H3K27M mutation with loss of H3K27me3	H3.3 K27M mutation confirmed by Sanger sequencing.
DNA methylation classifier	DMG, H3K27-altered methylation class	DKFZ classifier: DMG, H3K27-altered (calibrated score 0.98)
Additional molecular findings	Variable (TP53, ATRX, PDGFRA alterations common)	*Not methylated MGMT*, retained ATRX expression, Tumor cell positivity for p53*, TERT wt.*
Clinical course	Highly aggressive, median OS 9–15 months	Good postoperative clinical and neurological recovery, followed by adjuvant radiotherapy with 60 Gy in 30 fractions. The patient was subsequently enrolled in a clinical trial investigating ONC201 and remains under regular clinical and radiological follow-up at 7 months after surgery

In cases similar to the one described in this study, histologic and radiologic features may strongly suggest glioblastoma, IDH-wildtype, particularly when necrosis, microvascular proliferation, and high mitotic activity are present (7). Reliance on histopathology alone may therefore lead to diagnostic misclassification. Biologically, the H3K27M mutation results in the substitution of lysine with methionine at position 27 of histone H3, impairing PRC2-mediated trimethylation of H3K27 and leading to global epigenetic dysregulation, chromatin remodeling, and the maintenance of stem-like transcriptional programs. This epigenetic reprogramming underlies the distinct biological identity of H3K27-altered gliomas and distinguishes them from conventional IDH-wildtype glioblastomas.

Our case emphasizes the crucial role of integrated molecular diagnostics. Genome-wide DNA methylation profiling demonstrated a high-confidence match with the H3K27-altered methylation class, and Sanger sequencing confirmed the presence of the H3.3 K27M mutation. Importantly, real-time nanopore-based methylation profiling yielded concordant classification within 5 minutes, highlighting the feasibility of rapid, clinically actionable epigenetic diagnostics. Copy number analysis and targeted next-generation sequencing further refined the tumor’s molecular landscape.

Although a KRAS p.Gly12Ala (G12A) variant was identified, this alteration is not currently considered a therapeutically actionable KRAS alteration in this clinical and molecular context, and no approved or established RAS-targeted therapy was considered appropriate. Therefore, the identification of this variant did not provide a rationale for the use of a RAS inhibitor in the present case.

Collectively, these findings demonstrate that patient age and tumor location should not preclude testing for H3K27 alterations. Although non-midline H3K27-altered diffuse gliomas appear to be rare, their true incidence in adults may be underestimated, as comprehensive molecular profiling is not routinely performed in all glioma cases. Consequently, some tumors diagnosed as conventional IDH-wildtype glioblastomas in adults may in fact represent misclassified H3K27-altered gliomas. Integrated histo-molecular evaluation is therefore essential to achieve diagnostic precision, guide prognostic stratification, and identify patients who may benefit from molecularly targeted therapeutic trials, such as those involving ONC201. Molecular characterization may help identify patients who could be considered for enrollment in clinical trials evaluating investigational therapies. In the present case, the identification of an H3.3 K27M mutation contributed to the clinical rationale for considering enrollment in an ONC201 clinical trial. However, the presence of an H3.3 K27M alteration alone should not be interpreted as predictive of response to ONC201, particularly in an atypical non-midline tumor. The patient was subsequently enrolled in a clinical trial evaluating ONC201 in H3 K27M-mutant diffuse glioma (26).

Our case further highlights the value of combining morphology, methylation profiling, and rapid sequencing technologies in the diagnostic workup of atypical adult gliomas.

## Data Availability

The raw data supporting the conclusions of this article will be made available by the authors, without undue reservation.
